# ﻿Morphological analysis of regenerated antennae in the isopod *Porcellioscaber* (Isopoda, Crustacea), with emphasis on the main sensory structures

**DOI:** 10.3897/zookeys.1225.118414

**Published:** 2025-02-05

**Authors:** Primož Zidar, Primož Mihelič

**Affiliations:** 1 Department of Biology, Biotechnical Faculty, University of Ljubljana, Večna pot 111, SI-1000 Ljubljana, Slovenia University of Ljubljana Ljubljana Slovenia

**Keywords:** Aesthetascs, apical organ, autotomy, moulting cycle, regeneration, SEM, tricorn sensilla, woodlice

## Abstract

The second antennae, an important sensory organ of terrestrial isopods, are often attacked and amputated by predators or members of the same species. If an amputation does happen, the antenna usually regenerates after the very first or the next moult that follows. However, the new regenerated antenna is smaller than the original one. This raises the question of whether a smaller regenerated antenna is potentially also less functional as the sensory elements of the antenna undergo regeneration as well. In this study, the regeneration process of the second antenna of *Porcellioscaber* was followed after two consecutive amputations. The original antennae were compared with the regenerated ones under light and scanning electron microscopes in view of the dimensions of segments and the size and number of sensory elements on them. As expected, all regenerated antennae were reduced in size, however, the reduction of different antennal segments was different. The second segment of the flagellum was reduced by almost half as much as the first segment of the flagellum or the last segment of the peduncle. After the next consecutive amputation, the reduction of the regenerated antennae does not increase further. The size and shape of the apical organ and lateral seta did not change during the regeneration process, neither after the first nor after the next amputation. In contrast, the number of plural receptor elements, such as aesthetascs and tricorn sensilla, decreases in accordance with reduced segment size. Therefore, since only the number of the most common sensory structures is reduced during regeneration, the regenerated antenna probably retains its sensory functionality.

## ﻿Introduction

Epimorphic regeneration, by which an animal restores damaged or lost body parts, is a characteristic of many arthropods (reviewed in Hopkins and Das 2015). Many of them can regenerate limbs that were lost after injury or amputation, but not many can do that as adults (reviewed in Maruzzo and Bortolin 2013). Namely, a limb cannot be regenerated into a functional structure without undergoing one or more moults and many crustaceans, like most decapods, amphipods and isopods, moult throughout their life, however, the frequency of moulting may change with age (Skinner 1985).

*Porcellioscaber* (Latreille, 1804) is a common terrestrial isopod (Isopoda, Oniscidea) native to continental Europe, but distributed on all continents except Antarctica (Riggio 2013). In its environment, it may be attacked by predators or members of its own species and their second antennae can easily be damaged or lost (Schmalfuss 1998). Namely, as in other members of suborder Oniscidea, the second antenna of *P.scaber* is much larger than the extremely diminished first antenna and is also the most exposed limb (Schmalfuss 1998). The second antenna of *P.scaber*, as in most terrestrial isopods, consists of a five-jointed peduncle and a two-jointed flagellum and is considered the main sensory organ (Hoese 1989; Schmalfuss 1998). They possess several different sensory structures: an apical organ with a mechano- and chemoreceptory function (Mead et al. 1976), several aesthetascs with a chemoreceptory function (Hoese 1989) and many tricorn sensilla with a potentially hygroreceptory (Price and Holdich 1980) and/or mechanoreceptory function (Friedlander 1964; Ziegler and Altner 1995). The loss of the second antenna is undoubtedly a handicap for the animal due to its importance; therefore, the successful and rapid regeneration of the antenna is extremely important. Madhavan and Madhavan (1981) demonstrated that the amputation of one or both antennae of *Armadillidiumvulgare* (Latreille, 1804) in an earlier intermoult phase leads to a shortening of the moult cycle. Namely, moulting and regeneration are regulated by the same steroid hormones called ecdysteroids. Low levels of ecdysteroids observed after limb loss are thought to facilitate the initial stages of regeneration, while high levels inhibit it (Madhavan and Schneiderman 1969; Maruzzo and Bortolin 2013).

Regeneration of a damaged limb often begins with an autotomy of an injured part of the limb at a predetermined breakage point (PBP) proximal to the injury (Maruzzo et al. 2005; Hopkins and Das 2015). Autotomy minimises blood loss and ensures faster regeneration with fewer irregularities (Bliss 1960; Maruzzo et al. 2005). Klintz (1907) documented two such PBPs on the second antenna of *P.scaber*: one between the first and second peduncular segment and the other between the fourth and fifth peduncular segment. Which of the two PBPs was active depended on which segment the antenna was originally cut off. However, the regeneration of the last segment of the flagellum takes place without any triggered autotomy (Klintz 1907). In terrestrial isopods the regenerating structure develops within the antenna stub (Klintz 1907; Madhavan and Madhavan 1981), similarly as described in cockroach legs (Goss 1969; Maruzzo and Bortolin 2013). The regenerated limb first appears outside the stub after the very first or the next moult after amputation, all depending on the period of the intermoult cycle in which the limb was amputated (Madhavan and Madhavan 1981; Hopkins and Das 2015). According to Klintz (1907), the regenerated antenna is smaller, only 2/3 the length of the original one. This raises the question of whether a smaller regenerated antenna is potentially also less functional as sensory elements of the antenna undergo regeneration as well.

In this study, we aimed to determine the degree of reduction of the second antenna length and the number and size of antennal sensory elements in *P.scaber* after multiple amputations. We hypothesised that: (1) the regenerated antenna will be shorter after each successive amputation as limb regeneration requires a significant investment of energy (Hopkins and Das 2015); and (2) the number and size of antennal sensory elements will be reduced with the reduction of antennal segments as was reduced on regenerated legs in the amphipod *Parhyalehawaiensis* (Almazán et al. 2022). At the same time, by measuring the length of the moulting cycle, we tried to find out whether the length of the moulting cycle changes after limb amputation as reported for *A.vulgare* (Madhavan and Madhavan 1981).

## ﻿Materials and methods

### ﻿Experimental animals

Adult males of *P.scaber* of comparable size (10 ± 0.5 mm) were used in the study. All animals originated from the same laboratory-bred population. Due to sexual dimorphism, which is also reflected in the dimensions of the antennae (Lefebvre et al. 2000), females were excluded. The selected animals were kept in an incubator under the following conditions: 14 h day and 10 h night, 20 ± 0.5 °C, with prior gradual acclimatisation to these conditions. The humidity in the containers was maintained by hydrating the plaster that covered the bottom of the containers. Animals were fed with maple leaves and carrots. The animals were dorsally marked with a coloured permanent marker, which allowed us to easily notice when animals were moulted. The day after a successful moulting, the animals were marked again.

One day after moulting, the left second antennae of the animals were amputated in the middle of the third segment of the peduncle (P3) and fixed in 70% ethanol. In animals that successfully regenerated the amputated antennae, the regenerated antennae were re-amputated the day after moulting and fixed in the aforementioned manner. In animals that successfully regenerated the amputated antennae for a second time, the regenerated antennae were amputated again and fixed. In total, 11 original antennae (orig), 11 regenerated antennae of the first generation (reg1) and 9 regenerated antennae of the second generation (reg2) were analysed.

### ﻿Measurements

The animals were monitored for several months and the time between two successful moultings was recorded. The length of the moulting cycles of control animals, whose antennae were not amputated, were compared with the length of the moulting cycles of animals with amputated antennae. Some animals with amputated antennae were observed daily with a stereo microscope (Leica EZ4, Leica Microsystems, Germany) and all morphological changes at the amputation site were documented.

All amputated antennae were first analysed with a stereo microscope (Leica MZ FLIII, Leica Microsystems, Germany) using the Leica application suite (LAS) software (Leica Microsystems, Germany) for image capture. The maximum length and width of the last three segments of the antennae, that is, the 5^th^ segment of the peduncle (P5) and the first (F1) and second segment of the flagellum (F2), were measured using the Fiji image processing package of ImageJ2 (Schindelin et al. 2012). The length of P5 and F1 was measured between two joints, and the length of F2 between the joint and the base of the apical organ. The width was measured at the widest part of the segment. The 4^th^ segment of the peduncle (P4) was used for manipulation of the antenna and to attach it on a holder for SEM analysis.

**Figure 1.**
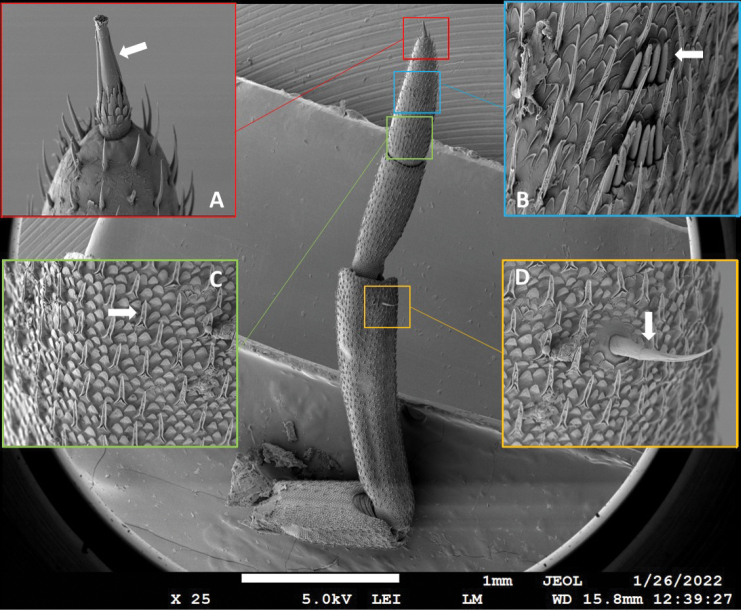
Sense organs on the second antenna in *Porcellioscaber***A** apical organ (arrow) **B** aesthetascs (arrow) **C** tricorn sensillum (arrow) **D** lateral seta on the fifth peduncular segment (arrow).

The amputated antennae (orig, reg1 and reg2) of seven males were dried with hexamethyldisilazane (HMDS, Merck, Germany). The individual antenna was attached to a metal holder (SPI supplies, USA) using adhesive aluminium foil (Fig. 1). This allows a greater rotation of the sample in the microscope. The samples were coated with platinum (up to 14 nm) with a sputter coating machine (SCD 050, BAL-TEC, USA). Such prepared samples were analysed with SEM (JEOL JSM-7500F, Japan). Images made with SEM were analysed in the ImageJ program with the Fiji package. The length of the apical organ (Fig. 1A), the number of aesthetascs (Fig. 1B) and the maximum density of tricorn sensilla on the lateral side of F2 (Fig. 1C) were analysed. The number of tricorn sensilla was determined by fitting the maximum number of sensilla bases into a square with sides of 100 µm (0.01 mm^2^). Additionally, the length of the seta on the lateral side of the fifth segment of the peduncle (P5 seta) (Fig. 1D) was analysed as well.

### ﻿Statistics

The Wilcoxon signed ranks test for dependent samples was used to compare the parameters measured on the original and regenerated antennae. Namely, we assumed that in an animal with a relatively shorter and narrower original antennae, the regenerated antennae are probably relatively shorter and narrower as well. We assumed the same for the sensory structures on the antennae. The length of the moulting cycle of control animals and the length of the moulting cycle after antennal amputation were compared using the Mann-Whitney test. The correlation between the parameters was determined using the Spearman correlation coefficient. All analyses were performed with IBM SPSS Statistics for Windows, v. 28.0.

## ﻿Results

### ﻿Pattern of antennae regeneration and the length of the moult cycle

**Figure 2.**
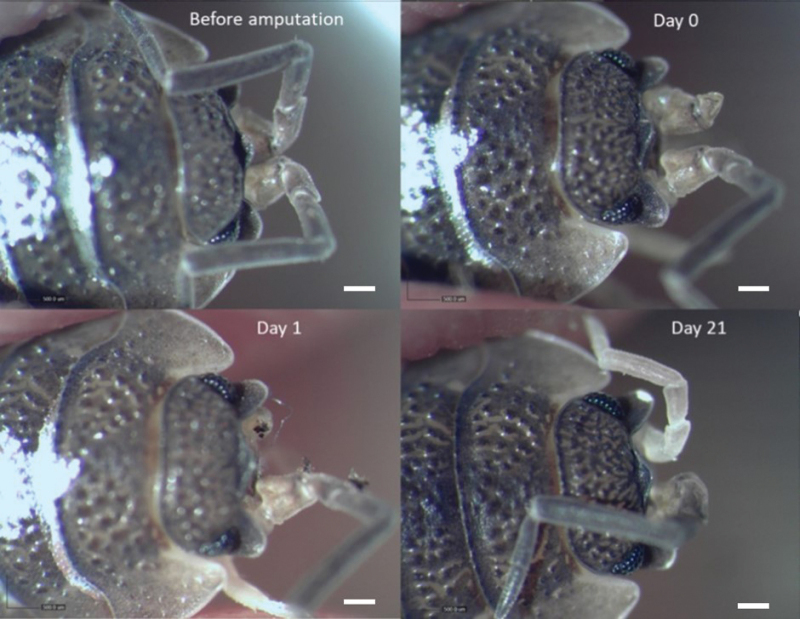
The regeneration process of the second antennae after amputation in *Porcellioscaber*. Before amputation: The animal with undamaged antennae; Day 0: The left antenna amputated in the middle of the third segment; Day 1: The left antenna after autotomy; Day 21: The regenerated left antenna. Scale bar: 0.5 mm.

In less than a minute after amputation of the antenna, the flow of hemolymph from the wound had stopped. In the next 24 h, autotomy occurred in 100% and 80% of the cases after the first and second consecutive amputation, respectively (Fig. 2). The PBP was at the junction between the first (basis) and second (coxa) segment of the peduncle. After autotomy, the wound on the basis segment was closed by a pigmented scab. In two animals (out of nine) autotomy did not occur after the second consecutive amputation, and a damaged third peduncular segment remained on the antenna residue until moulting. In all cases, with or without autotomy, no other morphological changes of the antenna stump were noticed until moulting. The regenerated antenna, in all cases, contained all segments but was less pigmented and visibly smaller compared to the other, undamaged antenna.

The length of the moult cycle did not differ between control animals and amputees (Mann-Whitney test, *p* = 0.321). The moult cycle lasted 16–32 days in animals without amputation (AVR = 24 days, SD = 5, *N* = 10) and 17–36 days in amputees (AVR = 26 days, SD = 5, *N* = 25).

### ﻿Dimensions of the second antennae

**Figure 3.**
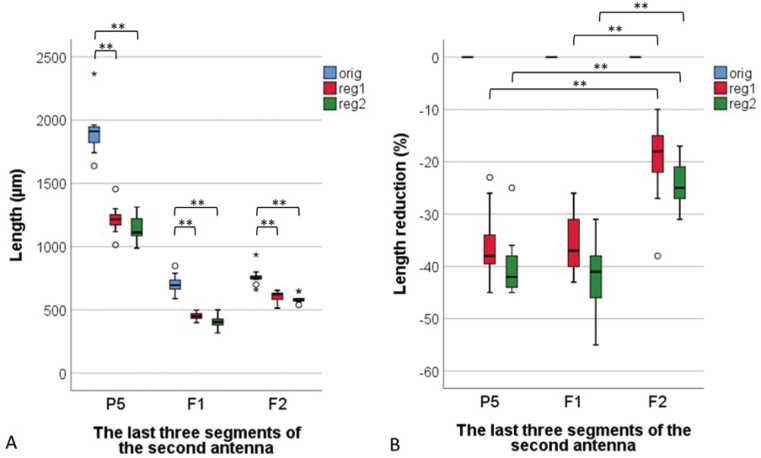
Length (**A**) and length reduction (**B**) of the last three segments (P5, F1 in F2) of the original antennae (orig), regenerated antennae after first regeneration (reg 1) and regenerated antennae after the second regeneration (reg2) of *Porcellioscaber*. Key: box: 25^th^, 50^th^, and 75^th^ percentile; whiskers: value ≤ 1.5 IQR (interquartile range); o – outlier: 3 IQR ≤ value > 1.5 IQR; * – extreme: value > 3 IQR; ** – significantly different, p < 0.01.

The measured part of the regenerated antennae was compared to the original ones, on average 32% and 37% shorter after the first and second regeneration, respectively. All three measured antennal segments were significantly shorter in all regenerated antennae (Wilcoxon signed ranks test, *p* < 0.01) (Fig. 3A). There were no significant differences in the length of the segments between the regenerated antennae of the first and second regeneration (reg1 and reg2; Wilcoxon signed ranks test, *p* > 0.069).

The reduction of the seventh flagellar segment (F2) was significantly (Wilcoxon signed ranks test, *p* < 0.01) smaller compared to the fifth and sixth segments (P5 and F1). After the first regeneration, P5 and F1 shortened by about 35%, while F2 only shortened by about 20%. After the second regeneration, P5 and F1 shortened by about 40%, while F2 only shortened by about 24%.

**Figure 4.**
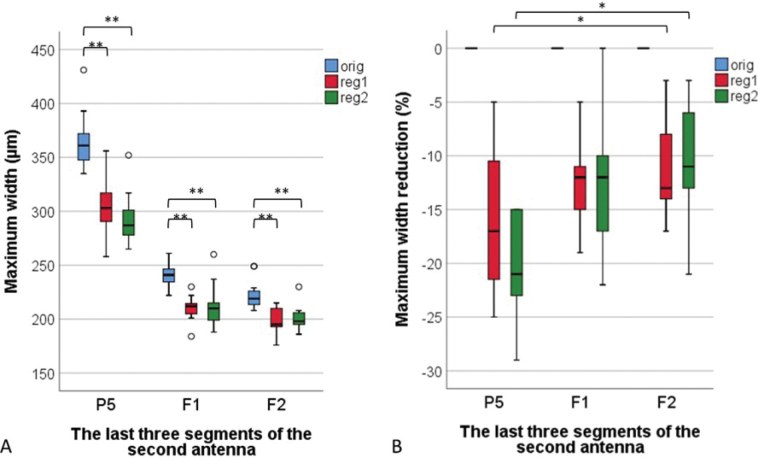
Width (**A**) and reduction of width (**B**) of the last three segments (P5, F1 in F2) of the original antennae (orig), regenerated antennae after first regeneration (reg 1) and regenerated antennae after the second regeneration (reg2) of *Porcellioscaber*. Key: box: 25^th^, 50^th^, and 75^th^ percentile; whiskers: value ≤ 1.5 IQR; o – outlier: 3 IQR ≤ value > 1.5 IQR; * – significantly different, p < 0.05; ** – as previous, but p < 0.01.

Individual segments of the regenerated antennae were significantly narrower than the original ones (Wilcoxon signed ranks test, *p* < 0.01) (Fig. 4A). There were no differences in segments’ width between the regenerated antennae of first and second regeneration.

The width reduction was the highest in P5 after the second regeneration (Fig. 4B). The width reduction of P5 was significantly higher only compared to that of F2 (Wilcoxon signed ranks test, *p* < 0.05).

### ﻿Analysis of sense organs

#### ﻿Apical organ

The apical organ of all regenerated antennae had a characteristic structure (Fig. 1A): the basal part arising from the indentation of the distal part of the last flagellar segment and continuing into the conical apical part with a tuft of short terminal hairs and two lateral sensilla lying close to the apical part.

**Figure 5.**
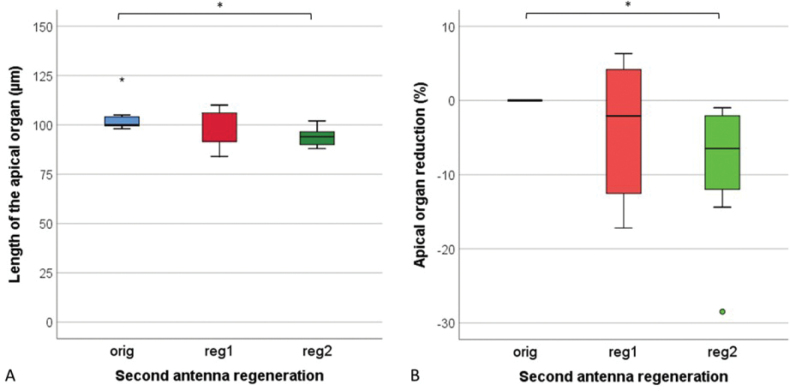
Length (**A**) and length reduction (**B**) of the apical organ on the last segment of the original antennae (orig), regenerated antennae after first regeneration (reg 1) and regenerated antennae after second regeneration (reg2) in *Porcellioscaber*. Key: box: 25^th^, 50^th^, and 75^th^ percentile; whiskers: value ≤ 1.5 IQR; o – outlier: 3 IQR ≤ value > 1.5 IQR; * – extreme: value > 3 IQR; * – significantly different, *p* < 0.05).

After the first regeneration, the length of the apical organ on the regenerated antennae (reg1) did not differ from the original length of the organ (Fig. 5). In three animals, the apical organs were a few percent longer (Fig. 5B). After the second regeneration (reg2), the newly formed apical organs were shorter than the originals in all animals (Wilcoxon signed ranks test, *p* < 0.05). On average, the length of the apical organs after the second regeneration decreased by 9% (Fig. 5B).

#### ﻿Aesthetascs

**Figure 6.**
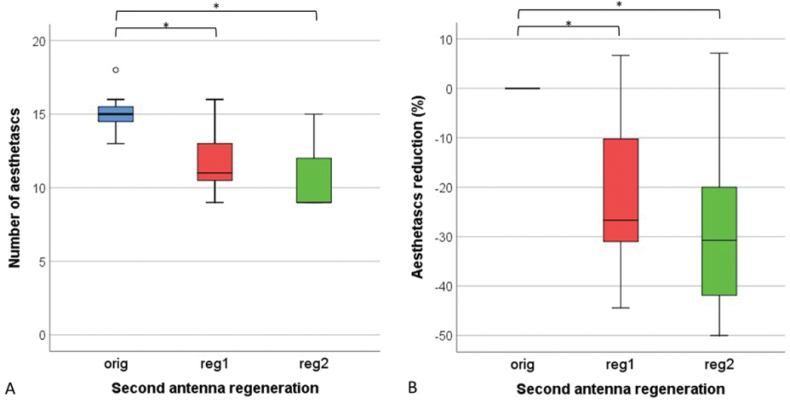
Number of aesthetascs (**A**) and aesthetascs number reduction (**B**) on the last segment of the original antennae (orig), regenerated antennae after first regeneration (reg 1) and regenerated antennae after second regeneration (reg2) in *Porcellioscaber*. Key: box: 25^th^, 50^th^, and 75^th^ percentile; whiskers: value ≤ 1.5 IQR; o – outlier: 3 IQR ≤ value > 1.5 IQR; * – significantly different, *p* < 0.05).

Aesthetascs on the last flagellar segment of the antennae (F2) were present in 2–4 groups of 2–6 units (12 in one case) and individually, up to 4 units per segment. The number and spatial distribution of aesthetascs on F2 differed between animals as well as between original and regenerated antennae of the same animal (Fig. 6A). On the regenerated antennae there were significantly (Wilcoxon signed ranks test, *p* < 0.05) fewer aesthetascs than on the original antennae (Fig. 6B). On average, the number of aesthetascs after the first and second regeneration decreased by 21% and 28%, respectively. However, in two cases the number of aesthetascs increased after regeneration.

#### ﻿Tricorn sensilla

**Figure 7.**
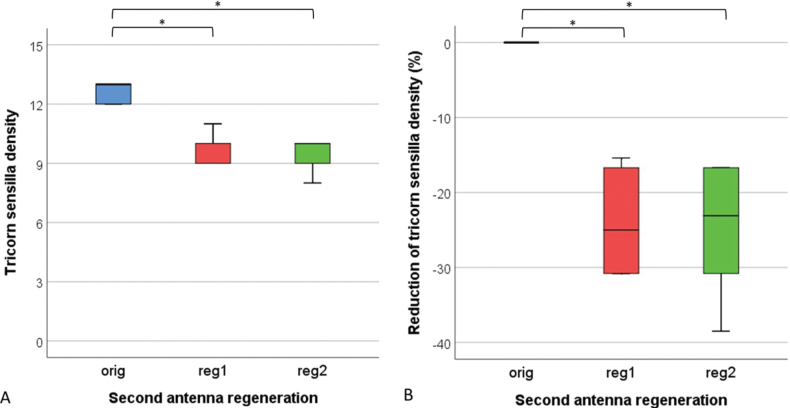
Tricorn sensilla density as number per 0.01mm^2^ (**A**) and reduction of tricorn sensilla density (**B**) on the last segment of the original antennae (orig), regenerated antennae after first regeneration (reg 1) and regenerated antennae after second regeneration (reg2) in *Porcellioscaber*. Key: box: 25^th^, 50^th^, and 75^th^ percentile; whiskers: value ≤ 1.5 IQR; o – outlier: 3 IQR ≤ value > 1.5 IQR; * – significantly different, *p* < 0.05).

The maximal density of tricorn sensilla on the last segment of the second antenna was 13 sensilla per 0.01 mm^2^ (Fig. 7A, orig). The density of sensilla on the regenerated antennae (reg1 and reg2) was significantly lower (Wilcoxon signed ranks test, *p* < 0.05) (Fig. 7A). The density of tricorn sensilla after the first and second regeneration did not differ (Fig. 7A, B). On regenerates the density decreased on average by 25% (Fig. 7B).

#### ﻿Lateral seta

**Figure 8.**
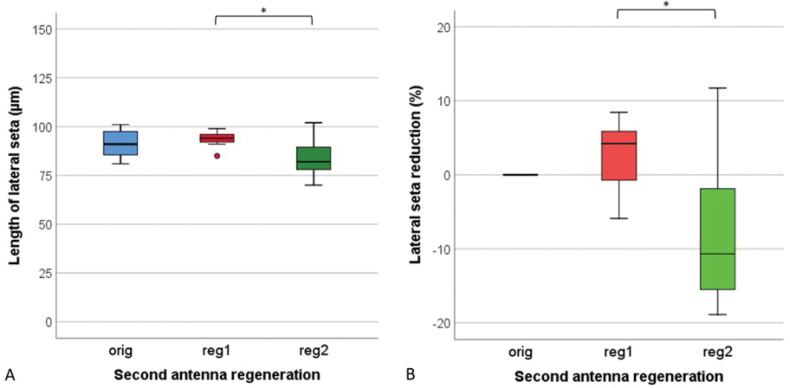
Length (**A**) and length reduction (**B**) of the lateral seta on the fifth peduncular segment of the original antennae (orig), regenerated antennae after first regeneration (reg 1) and regenerated antennae after second regeneration (reg2) in *Porcellioscaber*. Key: box: 25^th^, 50^th^, and 75^th^ percentile; whiskers: value ≤ 1.5 IQR; o – outlier: 3 IQR ≤ value > 1.5 IQR); * – significantly different, *p* < 0.05).

The length of the lateral seta on the fifth segment of the antennal peduncle of control animals was 81–101 µm (Fig. 8A). The seta has an articulated base, a surface with longitudinal ridges (looks striated), a curved posterior end and an annulus on an anterior third of the length (on Fig. 1D visible near the arrow). After the first regeneration the lateral seta did not differ from that of the original antenna neither by size nor by shape (Fig. 8A). In most of the regenerates, the seta was a few percent larger (Fig. 8B). In one animal, after the first regeneration, two setae were present: in addition to the lateral seta of normal dimensions, a similar but somewhat shorter seta was present. After the second regeneration, this animal, like the others, had only one lateral seta once again. After the second regeneration, the seta was shorter in 5 out of 9 animals, for 7% up to 19%.

## ﻿Discussion

### ﻿Pattern of antennae regeneration and the length of moult cycle

The autonomy of the part of the damaged antenna was the first noticeable change in the process of regeneration. Autonomy occurs within 24 hours after amputation between the first and second segment of the antenna (in 90% of cases). This PBP site was first documented by Klintz (1907). The process of autotomy happened overnight, so we don’t know if other limbs may be involved in this process, as in autotilly (Maruzzo et al. 2005). After removing a similar number of segments, autotomy at the same PBP also occurred in *A.vulgare*, but in a smaller proportion (Madhavan and Madhavan 1981). As mentioned in the review article of Maruzzo et al. (2005), the PBP in the legs of *Porcellio* sp. contains a connective tissue septum called the autotomy membrane, which acts as a valve that closes the hole left after autotomy, minimising the wound trauma (Hopkins and Das 2015). Whether the PBPs of the antennae of *Porcellio* also contain the autotomy membrane is still to be investigated. Namely, in the genus *Asellus*, the autotomy membrane is present in the PBP of the legs but not in the antennae (Needham 1965; Maruzzo et al. 2005). A day after the autotomy, we noticed rather a large, pigmented scab covering the wound. The pigmented scab likely resulted from the action of the immune system: degranulation of hemocytes (Hopkins 1993) and activation of the phenoloxidase system, which initiates melanization (Theopold et al. 2004). We observed no other changes in the limb stub, while Madhavan and Madhavan (1981) reported that in *A.vulgare*, the distal surface of the wound becomes whitish due to the withdrawal of the pigmented epithelium. In *P.scaber* and *A.vulgare* a regenerating antenna was produced entirely inside the limb stub (Madhavan and Madhavan 1981), in our case solely in the small first segment - basis. Therefore, the space for a regenerating antenna was extremely small. A cuticular sac at the tip of the stub, where a regenerating limb is produced, was reported for decapod crustaceans (Maruzzo and Bortolin 2013) as well as for *Asellus* (Needham, 1965 in Maruzzo et al. 2005) but not for terrestrial isopods. The regenerated antennae were smaller and whitish compared to contralateral antennae as observed also by Klintz (1907) and Madhavan and Madhavan (1981). However, the regenerated antennae show the same properties as the original one in terms of the ability to repeat autotomy and regenerate as described for legs in fiddler crab *Ucapugilator* (Hopkins 1993).

After amputation of the antenna, the length of the moulting cycle was, on average, two days longer than that of control specimens, but the difference was not statistically significant. In contrast, in *A.vulgare* the moulting cycle was shortened by an average of three days when one antenna was amputated two days after moulting (Madhavan and Madhavan 1981). According to Madhavan and Schneiderman (1969), arthropods require some basic amount of ecdysteroid hormones for regeneration to begin. As reported by Hoarau and Hirn (1981) amputation of a limb causes a decline in ecdysteroids in the hemolymph and takes time to return to the baseline, causing the moulting cycle to lengthen. It seems that the low level of ecdysteroids in the hemolymph of our animals the day after moulting was further reduced by the amputation, which caused a delay in moulting.

### ﻿Reduction of the antennae and sensory structures during the regeneration process

The measured part of the regenerated antennae of our animals was about one-third shorter than the same part of the original antennae, which agrees with the findings of Klintz (1907). The reduction of the regenerated antenna could be explained by the additional expenditure of energy and material for the regeneration process, but more likely by the limited space in which the regenerated limb is formed (Hopkins and Das 2015). We found that even if the antennae are amputated several times in a row, there are no major differences between the dimensions of the regenerated antennae. However, neither energy expenditure nor limited space can explain why the reduction of individual segments of the antennae in the regeneration process is different. Namely, the shortening of the most distal antennal segment was almost half that of the fifth or sixth segment. It can be assumed that the last flagellar segment is functionally more important than the other segments of the antenna, as it carries many sensory organs.

The morphology of the regenerated apical organs did not differ from the original ones and, in all cases, corresponded to the description of the organ given by Hoese (1989). Besides, no length reduction of the apical organ was observed until the second regeneration (Fig. 5B, ‘reg2’). Although the last segment of the flagellum shortened during the second regeneration on average by a quarter (Fig. 3B, ‘reg2’), the shortening of the apical organ did not exceed 10%. Apical organ length showed only a weak and insignificant correlation with the length (*r_s_* = 0.399, p = 0.073) or width (*r_s_* = 0.337, p = 0.136) of the last flagellar segment. This suggests a key role of the apical organ in isopod chemo- and mechanoreception. Namely, the apical organ may contain tens of receptor cells (Seelinger 1977) that respond to mechanical, olfactory, and gustatory stimuli and thus play an important role in surface screening (Hoese 1989), social interactions (Linsenmair and Linsenmair 1971) and food detection (Seelinger 1977).

Similar to the apical organ, no significant reduction in length or change in shape of the lateral seta was noticed. The up to 100 µm long seta was located on the lateral side of the last segment of the peduncle on all antennae, original and regenerated. A similar seta with two segments and a striated surface was described on the antennal peduncle of *A.vulgare* by Rife (1993). This seta probably has a mechanoreceptory function (Crouau, 1995, 1997). Despite around 40% reduction in the length of the last peduncle segment and around 20% reduction in its width, the seta length reduced insignificantly. Its length showed only a weak and insignificant correlation with the length (*r_s_* = 0.268, *p* = 0.241) and with the width (*r_s_* = 0.390, *p* = 0.080) of the last peduncular segment.

In accordance with the reduction of the antennal segments’ surface, the number of plural sensory organs, such as aesthetascs and tricorn sensilla, is reduced. The number of aesthetascs decreased mainly due to the decrease in the number of aesthetascs per group and not by reduction of the number of groups. Aaesthetascs were mainly in 3–4 groups as reported by Hoese (1989). However, up to 4 aesthetascs were frequently located also individually outside these groups. Reduction of aesthetascs coincides with the reduction of the length and width of the last flagellar segment. Spearman’s correlation coefficient showed a moderate to strong positive correlation between the number of aesthetascs and the length (*r_s_* = 0.671, *p* = 0.001) and width of the segment (*r_s_* = 0.735, *p* = 0.000). Aesthetascs have an important olfactory role (Hoese 1989; Schmalfuss 1998).

During the regeneration, the density of tricorn sensilla on the last segment of the flagellum decreased by around 25%. Spearman’s correlation coefficient showed a strong positive correlation between the density of tricorns and the length of the segment (*r*_s_ = 0.784, *p* = 0.000) and a moderate correlation with its width (*r*_s_ = 0.557, *p* = 0.009). Tricorn sensilla are characteristic for terrestrial isopods and are the most numerous sensilla on the second antennae of terrestrial isopods (Holdich 1984; Schmalfuss 1998). These triangular-shaped structures are known to contain sensory cells (Ziegler and Altner 1995), but their role is still unknown. It was suggested that they may have a hygroreceptory (Price and Holdich 1980), mechanoreceptory (Holdich and Lincoln 1974) or even chemoreceptory function (Tsuneo 1989).

## ﻿Conclusions

This study revealed that:

- The length of the moult cycle did not differ between amputated and control individuals.
- The regenerated antennae are reduced in size compared to the original ones, however, the reduction does not increase with multiple consecutive amputations.
- The reduction of individual antenna segments in the regeneration process is different. The most distal segment of the flagellum receives the smallest share of the reduction.
- The size and shape of the apical organ and lateral seta do not change during the regeneration process, although the size of the segment on which they are located decreases. In contrast, the number of plural receptor elements, such as aesthetascs and tricorn sensilla, decreases in accordance with the segment size reduction.

## ﻿Additional information

### Author ORCIDs

Primož Zidar https://orcid.org/0000-0002-9876-621X

### Data availability

All of the data that support the findings of this study are available in the main text.
